# Medical News and Miscellany

**Published:** 1891-11

**Authors:** 


### Medical News and Miscellany.

Dr. J. D. Wingate has removed from Llano to Comanche, Texas.

Dr. A. C. Walker, of Rockdale, is at the New York Polyclinic, and will supply the JOURNAL each month with interesting clinical notes from the great surgical centres.

Dr. M. A. Saylor, of Gonzales county, Pilgrim Lake, has sold his practice to Dr. H. H. Beverly, of that place, and is in Austin, resting, seeking, meantime, a new location.

Married.—At Dallas, November 4th inst., at the residence of the bride's parents, Dr. and Mrs. E. L. Thompson, Dr. W. A. Howard, of Waco, to Miss Fannie Thompson, of Dallas.

Dr. B. A. Harris, of Okla Union, Texas, is at St. Louis, attending a special course of lectures at the Marion-Sims Medical College. He renews his subscription, requesting the JOURNAL to be sent to St. Louis, and says "I can't do without the JOURNAL; you may put me down as a permanent subscriber."

Compressed Ophthalmic Discs.—The JOURNAL has before called attention to this new and elegant pharmacal aid to the doctor. Messrs. Wyeth & Co., Philadelphia, the manufacturers, will send samples to any one free, if the JOURNAL be mentioned. The discs are neat, cleanly and economical, and a big saving in the use of the expensive alkaloids used in treating diseases of the eye.

Feick Bros., of Pittsburg, Pa., are now offering their celebrated Aseptic pocket case, which has always sold for $11.00, for $9.75, for only thirty days, and this journal to be mentioned in the order. This is the neatest and most compact and elegant pocket case we have ever seen;—we having bought a good many of them for our readers—can testify to their excellence, and every doctor should now treat himself to one while they are offered so low.

Dr. T. W. Schlenker, of Ridgetown, Ontario, has come to Austin for his health. The doctor is an accomplished young physician, and finds a warm reception at the hands of the warm-hearted people of this sunny clime. He bears the seeds of the dread phthisis in his system, and his improvement since coming to Austin shows the wisdom of his move. Many persons, now useful citizens of Austin, came here with consumption, and are now cured. This speaks volumes for the pure air of Southwest Texas.

---

**Died.**—At his home near Willis Point, Texas, Sunday, November 1st, inst., after a brief illness, Dr. W. T. Strain.

Dr. Strain was a young man, not over thirty years of age, and a man of rare promise, as well as of unusual ability. His death was as unexpected as it was deplored. He had suffered a fall some time previously, but it was thought he had sustained no injury. In two weeks from date of fall, he had a chill, followed by another next day, and died at 2.30 p. m. Sunday, November 1st, it is supposed, of blood poisoning. Dr. Strain was a member of the Texas State Medical Association, and of the Terrell Medical Society.

---

"**The Climate of Southern California in relation to diseases,**" is the title of a very excellent paper, by Dr. W. A. Edwards, of San Diego, California, who has a sanitarium in that home of perpetual summer. The paper has appeared in the "*Climatologist*" and several other journals, and hence we can not republish it. To those in colder climates, especially those who suffer from pulmonary troubles, the paper will be of special value and interest. They should go to California (or come to Austin), and get well. Dr. W. A. Edwards, of San Diego, Cal., will gladly send a copy of the paper to any one, free, who will mention the Journal.

---

**Poeta Nascitur Non Fit.**—We have always held that the true surgeon is, like the poet, "*born* and not made." This reflection comes to us in reading the announcement that Dr. H. C. Rogers has recently located at Hot Springs, Ark. The Rogers family is a most remarkable one—a family of *born surgeons.* The father of the present generation—the two Doctors Rogers, W. B. of Memphis, and H. C., of Hot Springs,—was a most distinguished operator, and whether this talent "runs in the family" or not, both sons possess it in a marked degree. Dr. W. B. Rogers, at Memphis, who has a surgical infirmary, has made a record of which any man might well be proud. He is an occasional contributor to the JOURNAL, and his articles are always read with pleasure and profit.

---

Memorials of Gen. Lee.—Gen. Long's celebrated work: "Memorials of Robt. E. Lee," which had a big run at $3.75 a copy, can now be had for 50 cents a copy! Not a cheap edition, but the identical, handsome, cloth and gold edition. This can only be done, however, by taking the "*Cosmopolitan*," the leading and best literary magazine now published. The subscription price is only $3.00. The illustrations alone must cost a fortune, and we do not see how the publishers can afford it. The postage on the Memoirs, which you must pay, is 20 cents. So, send us $5.70 and you will get DANIEL'S TEXAS MEDICAL JOURNAL and the "*Cosmopolitan*" one year; and you will receive by mail, *free*, a copy of the Memorials of General Lee. This is an unprecedented offer, and is only temporary.

---

Solid Comfort and Safety.—Doctor, should you have occasion to go to St. Louis—go via Texarkana and take one of the Missouri Pacific and Iron Mountain Railway Co.'s splendid vestibule trains. This is not only the most direct and quickest route, but the service in every respect is high class, the coaches clean, commodious and comfortable, the officers—from General Passenger Agent H. C. Townsend, Esq., at St. Louis,—down to the brakemen, are courteous, and solicitous for the comfort and safety of passengers. Travel is robbed of its discomforts on this route, and it becomes simply a pleasure,—the route lying in and running through a beautiful, thrifty and prosperous section of country. For full information and maps address General Passenger Agent Townsend, St. Louis, mentioning the JOURNAL.

---

Treatment Wanted:—Dr. M. M. Gilbert, of Arizona, a subscriber of DANIEL'S TEXAS MEDICAL JOURNAL, asks our readers to give through the JOURNAL "the best treatment for screw worms in the nose," (in cattle, we presume,) and also "for itch in horses." The latter, the Doctor says, is a common and very annoying trouble in that country, and one which the veterinarians do not treat successfully.

Dr. F. A. Schmidt, of Schulenburg, Texas, published in the JOURNAL some years ago, an account of screw worms in the nose of a young person. Calomel, blown into the nostrils, dislodged them, and some antiseptic and cleansing wash followed, we believe. Let us hear from you, gentlemen, readers of the Red Back. You see, it is read away up in Arizona as well as everywhere else.

---

Appointment of Lecturers in Texas Medical College.—The Board of Regents have made the following appointments: Dr. R. C. Hodges, of Galveston, lecturer on the diseases of the eye; Dr. R. W. Knox, of Houston, lecturer on diseases the skin; Dr. H. P. Cooke, of Galveston, lecturer on diseases of children; Dr. Allen Smith, lecturer on nervous diseases, and Dr. Ed. Randall, lecturer on diseases of the chest. These are all excellent appointments, and with the very able faculty gives the College a crops of active workers not surpassed by any college in America.

The class numbered twenty-two matriculants; but when we consider that a preliminary examination is required for matriculation—that the course consists of three lecture terms (graded) of seven months each, the high and thorough curriculum—the comparatively high fees—as high as the best—and then consider how much cheaper and easier graduation is at other relatively good schools, this is a very good beginning. The Texas College has set the mark high; and as its success does not depend on fees from students,—the professors being paid from $2,000 to $3,000 salary each—this standard will be strictly adhered to. It is the intention to make a diploma from the Medical College of the Texas University *mean something*, and to have a value. The management are determined to make it a medical school second to none in the western world.

